# Correlation between mental health literacy, communication anxiety, and mobile phone dependence among first-year college students

**DOI:** 10.1186/s12889-026-28594-5

**Published:** 2026-07-20

**Authors:** Jin-mei Yang, Mi-zhen Ye, Fen-xin Wu, Si-yang Ye

**Affiliations:** 1 School of Public Health, Guangdong Medical University, Dongguan, Guangdong 523808 China; 2 The Second Affiliated Hospital of Guangdong Medical University, Zhanjiang, Guangdong 524003 China

**Keywords:** College students, Mental health literacy, Communication anxiety, Mobile phone dependence

## Abstract

**Objective:**

This study aimed to explore the relationship between mental health literacy, communication anxiety, and mobile phone dependence among first-year medical students.

**Methods:**

This study adopted a cross-sectional design. A total of 547 valid questionnaires were included in the analysis. Mobile phone dependence, mental health literacy, and communication anxiety were assessed using the Smartphone Addiction Scale–Short Version (SAS-SV), Mental Health Literacy Questionnaire–Short Version for Adults (MHLq-SVa), and Interaction Anxiousness Scale (IAS), respectively. Spearman’s rank correlation and hierarchical regression analyses were conducted to examine the associations among mental health literacy, communication anxiety, and mobile phone dependence.

**Results:**

Mobile phone dependence was identified in 64.9% of first-year college students. Univariate analysis showed that mobile phone dependence was significantly associated with communication anxiety and the duration of mobile phone use (*P* < 0.001). Hierarchical regression analysis, after adjusting for demographic covariates, indicated that communication anxiety was significantly and positively associated with mobile phone dependence (*β* = 0.380, *P* < 0.001). Additionally, monthly living expenses were significantly positively associated with mobile phone dependence (1000–1999 Yuan: *β* = 0.158, *P* = 0.016; ≥2000 Yuan: *β* = 0.181, *P* = 0.007). Conversely, mental health literacy was not significantly associated with mobile phone dependence (*β*=−0.027, *P* = 0.492).

**Conclusion:**

Mobile phone dependence among first-year college students was associated with monthly living expenses and communication anxiety, whereas no significant association was found with mental health literacy. These findings suggest that communication anxiety may warrant greater attention than mental health literacy in future intervention research addressing mobile phone dependence in this population.

**Supplementary Information:**

The online version contains supplementary material available at https://doi.org/10.1186/s12889-026-28594-5.

## Introduction

First-year post-secondary students are at a critical stage of transitioning from adolescence to adulthood. With the transformation of learning modes and living environments, students entering college are faced with increased pressure that makes them more at risk of mental health problems [1].Mobile phone dependence—also known as mobile phone addiction, mobile phone use disorder, or problematic mobile phone use—refers to excessive or inappropriate mobile phone use [2]. It has an adverse impact on individual social and psychological functioning [3].

By June 2024, the number of mobile internet users in China had reached 1.096 billion [4]. It is worth noting that as the core group of Internet users, the frequency and duration of mobile phone use of college students are at a high level, and the comprehensive prevalence of mobile phone addiction is increasing year by year [5]. In addition, it has been found that mobile phone dependence is closely related to a variety of mental health problems in college students [6–10], among which the risk of severe depression and anxiety in mobile phone dependence is significantly higher than that in non-dependence, and there is a significant positive correlation between them. Mental health literacy refers to the knowledge, attitudes, and behavior of individuals in the process of promoting their own and others’ mental health and in coping with their own and others’ mental illnesses [11]. Mental health literacy is a key ability that allows individuals to identify, manage, and prevent mental disorders, and its level is closely associated with mental health status [11]. First-year college students with low mental health literacy lack the ability to identify their own psychological problems, and do not have effective emotion regulation and stress-coping skills. When facing difficulties in adapting to pressure and interpersonal communication, it is difficult to relieve negative emotions in a reasonable way, and may use mobile phones as a way to escape realistic pressure and relieve negative emotions. By excessive use of mobile phones to obtain short-term psychological comfort, it is easy to fall into the dilemma of mobile phone dependence in the long run [12–14]. On the contrary, a high level of mental health literacy can help to better be aware of their emotional state, master effective emotional regulation methods, reduce excessive dependence on mobile phones, and thus reduce the risk of mobile phone dependence. Existing studies mostly focus on the direct correlation between mobile phone dependence and various psychological problems, but rarely explore the role of mental health literacy, a protective factor, in the relationship between mobile phone dependence and psychological problems of first-year college students, and have not clearly examined its potential role in preventing mobile phone dependence [15–17]. Communication anxiety, a form of social anxiety related to interpersonal communication, is a common mental health problem. It refers to the emotional reactions, such as worry, tension, and fear, generated by the interpersonal communication environment [18, 19].

From the perspective of behavioral decision theory, first-year college students with higher levels of communication anxiety are more likely to experience negative emotions in real interpersonal communication and have an obvious tendency of social avoidance. As an indirect, non-face-to-face communication tool, mobile phone can effectively reduce the tension and discomfort in interpersonal communication, and become an alternative way for such individuals to escape from reality and meet their social needs [20–22]. Lai et al. [14, 23] found that communication anxiety was positively correlated with mobile phone dependence. However, it is not clear whether the mobile phone dependence, and the relationships among these three variables have not been adequately examined.

**Fig. 1:**
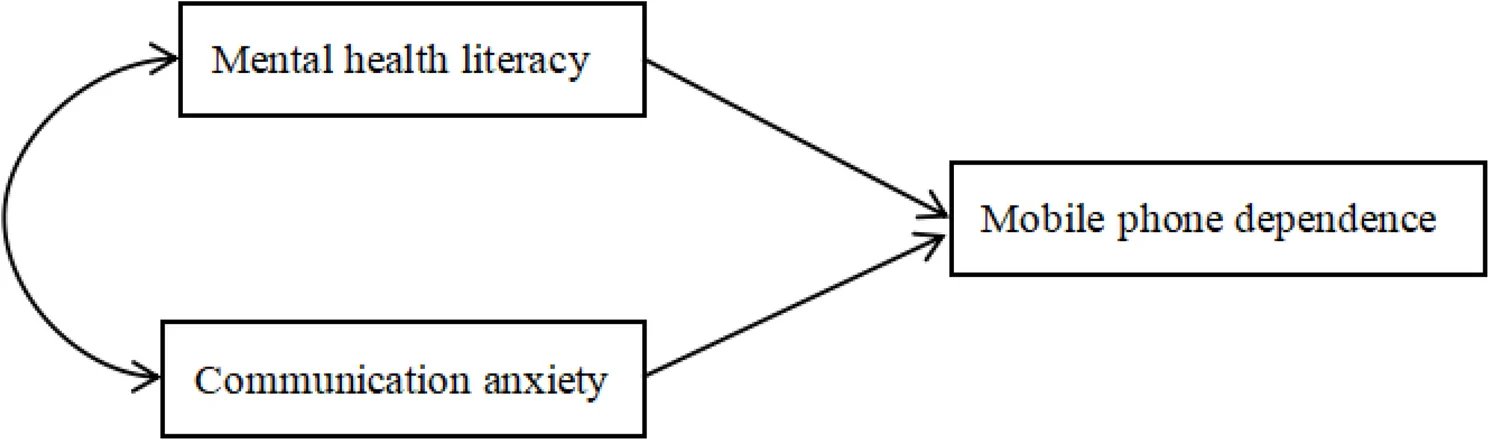
Conceptual framework of the study

Based on the above research background and gaps, this study proposes the following hypotheses: (1) Mental health literacy among first-year college students is negatively correlated with mobile phone dependence; (2) Communication anxiety among first-year college students is positively correlated with mobile phone dependence; (3) The positive association between communication anxiety and mobile phone dependence would be weaker among first-year college students with higher mental health literacy than among those with lower mental health literacy.Therefore, in this study, we selected first-year students at a medical university as the participants to analyze the relationship between mental health literacy, communication anxiety, and mobile phone dependence. The findings of this study may provide evidence for future research and intervention development related to mobile phone dependence and college students’ mental health. The conceptual framework of the study is presented in Fig. 1.

## Subjects and methods

### Subjects

In this study, all first-year students at a medical university were selected as the target population Participants were recruited on-site during class breaks in early September 2024. A QR code for survey access was presented to students who voluntarily expressed interest in participating, thereby achieving comprehensive coverage of the target population. On the premise of following the principle of informed consent, the two-dimensional code generated by the Credamo platform (https://www.credamo.com) was used to collect data by voluntarily filling out the questionnaire anonymously. We distributed 733 questionnaires and 547 valid questionnaires were retrieved, resulting in a response rate of 74.62%. Among the respondents, there were 240 males and 307 females. Due to the voluntary participation method, this study belongs to non-probability sampling. Inclusion criteria: (1) first-year undergraduates in the target medical college; (2) Complete the questionnaire and the completion time was ≥ 300 s; (3) No repeated submission was verified by Credamo platform through IP address and device identification code; (4) Informed consent. Exclusion criteria: after the questionnaire was collected, the data with abnormal response time and obvious inconsistencies or regular answers in the personal information options were excluded. This study has been approved by the Clinical Research Ethics Committee of the Affiliated Hospital of Guangdong Medical University (PJKT2023-158).

### Research instruments

#### Smartphone addiction scale – short version, SAS-SV

The SAS-SV scale, originally developed by Kwon et al. [24] in 2013, was translated into Chinese by Zhang et al. [25] in 2019. It is used to assess the degree of mobile phone dependence of individuals. A six-point Likert scale is utilized, with scores ranging from 1 to 6 as follows: 1 = Strongly Disagree, 2 = Moderately Disagree, 3 = Slightly Disagree, 4 = Slightly Agree, 5 = Moderately Agree, and 6 = Strongly Agree. The total score ranges from 6 to 60, with higher scores indicating a higher level of mobile phone dependence. Specifically, a score of ≥ 31 for males or ≥ 33 for females can be interpreted as mobile phone dependency. Based on the established psychometric properties in college student populations (Cronbach’s α = 0.90, test-retest reliability = 0.71), the SAS-SV was selected for this study. The 10-item abbreviated structure is well-suited for large-scale surveys, and its gender-specific diagnostic thresholds (≥ 31 for males, ≥ 33 for females) provide clear operational criteria for identifying mobile phone dependence [26]. The scale consists of 10 items, with a questionnaire Cronbach’s α coefficient of 0.863.

#### Mental health literacy questionnaire – short version for adults, MHLq-SVa

We also used the MHLq-SVa, developed by Luisa Campos et al. [27]. This scale comprises 16 items, organized into four dimensions: Mental health knowledge, Prejudice, Help-seeking behavior, and Self-help strategies, and employs a five-point scoring system. The total score ranges from 16 to 80, with higher scores indicating higher levels of mental health literacy. Prior research has demonstrated that the revised Chinese version of the Mental Health Literacy Questionnaire-Short Version for Adults (MHLq-SVa) maintains the same factorial structure as the original instrument, indicating adequate cross-cultural structural validity of the parent scale [28].The Cronbach’s α coefficient for the questionnaire is 0.870.

#### Interaction anxiousness scale, IAS

Finally, we employed the IAS, developed by Leary in 1983 [29]. Previous studies have established that the Chinese version of the Interaction Anxiousness Scale (IAS) serves as a valid instrument for assessing social anxiety among Chinese college students [30]. The scale employs a five-point Likert scoring system and consists of 15 items. Higher scores indicate a higher level of social anxiety among individuals. In this study, IAS scores ranging from 15 to 35 were classified as low interaction anxiety, scores from 36 to 55 as moderate-interaction anxiety, and scores from 56 to 75 as high interaction anxiety [31]. The Cronbach’s α coefficient for the questionnaire was 0.738.

#### Demographic characteristics

Demographic characteristics included gender, major (categorized as clinical medicine—including pediatrics, anesthesiology, or medical imaging—and non-clinical medicine), family residence, monthly living expenses, parental marital status, only-child status, and current romantic relationship status.Monthly living expenses were defined as the amount of money students received from their families or other sources to cover monthly living costs while attending school. The duration of mobile phone use was calculated based on Hamamura’s study [32]. The average daily the duration of mobile phone use was derived from respondents’ reported usage times on weekdays and weekends, according to the formula: Average Daily Usage Time = (Weekday Usage Time × 5 ་ Weekend Usage Time × 2) / 7. Unanswered items were considered missing responses, and the missing values were imputed using multiple imputation methods.

### Statistical methods

The data were analyzed using IBM SPSS 21.0 software. For the analysis of proportions, frequency counts, and composition ratios were employed to describe the data. The results of the Shapiro-Wilk test indicated that mental health literacy did not follow a normal distribution. Therefore, the data were described using the median and interquartile range (IQR). For the comparison of proportions, chi-square tests were employed. Post-hoc comparisons were conducted using the Bonferroni method. We also used hierarchical regression analysis to explore the factors associated with mobile phone dependence among college students. Four hierarchical regression models were specified: Model 1 (demographic characteristics), Model 2 (demographic characteristics and mental health literacy), Model 3 (demographic characteristics, mental health literacy, and communication anxiety), Model 4 (Model 3 plus the interaction term between mental health literacy and communication anxiety). The rationale for entering the variables in this sequence was to follow an analytical approach that progresses from general to specific and from basic to in-depth. This hierarchical modeling approach allowed the relative contributions of these variables to be evaluated. Prior to hierarchical regression analysis, the assumptions of linearity, homoscedasticity, residual normality, independence of errors, and multicollinearity were examined. No substantial violations were observed; all variance inflation factor values were below 2.817, tolerance values exceeded 0.1, and the Durbin-Watson statistic was 2.193. Missing data were observed for mobile phone use duration in one participant and were handled using the multiple imputation procedure in SPSS. The imputation model included all variables used in the main analyses. Five imputed datasets were generated and pooled according to Rubin’s rules. This approach assumes that the missing data mechanism was missing at random (MAR), as there was no evidence to suggest that the missingness was systematically related to the unobserved values themselves.

## Results

### Demographic characteristics of the survey participants

Among the survey participants, 355 (64.9%) students were identified as having mobile phone dependence. The prevalence of mobile phone dependence was 67.9% among male students and 62.5% among female students. This difference in gender was not statistically significant (*P* > 0.05). The total score for mental health literacy among college students was 70 (64,76). Among them, male students scored 69 (64,76), while female students scored 70 (65,75). No statistically significant difference was found between the two groups (*P* > 0.05). Demographic characteristics are detailed in Table 1.

**Table 1:** Demographic characteristics and scale scores of participants of Participants (*N* = 547)

Demographic characteristics	*n*	Proportion (%)	Total score of mental health literacy [Median (IQR)]	Mobile phone dependence [Median (IQR)]	Communication anxiety [Median (IQR)]
Gender					
Male	240	43.88	69(64,76)	35(29,49)	45(40,51)
Female	307	56.12	70(65,75)	35(29,41)	45(40,51)
Major					
Clinical medicine	495	90.49	70(64,76)	35(29,40)	45(40,51)
Non-clinical medicine	52	9.51	68(63,73)	35(28,39)	46(40,50)
Residential location of household					
Urban area	376	68.70	70(65,76)	35(29,41)	45(40,51)
Rural area	171	31.30	70(63,74)	35(28,40)	45(40,50)
Parental marital status					
Primary marriage	490	89.60	70(64,76)	35(29,40)	45(40,50)
Remarried or divorced	57	10.42	70(65,76)	37(30,41)	46(41,54)
Only child status					
Yes	121	22.10	69(66,76)	35(29,40)	44(40,50)
No	426	77.90	70(64,75)	35(29,40)	45(40,51)
Monthly living expenses (Yuan)					
< 1000	49	8.96	71(64,78)	27(20,41)	45(38,51)
1000–1999	390	71.30	69(64,74)	35(30,40)	45(41,51)
≥ 2000	108	19.74	72(65,77)	35(30,41)	45(39,50)
Current romantic relationship status					
Yes	53	9.69	70(65,76)	36(28,41)	43(37,47)
No	494	90.31	70(64,75)	35(29,40)	45(40,51)
The duration of mobile phone use (h)					
1-4.9	191	34.92	70(65,75)	33(28,40)	45(40,51)
5-9.9	294	53.75	70(64,76)	36(30,41)	45(40,57)
≥ 10	62	11.33	70(64,77)	36(29,41)	47(42,51)

### Univariate analysis of mobile phone dependence among college students

The results of the univariate chi-square test for mobile phone dependence among college students indicated that mobile phone dependence was significantly associated with communication anxiety and the duration of mobile phone use (*P* < 0.001). Detailed results are presented in Table 1. Post-hoc pairwise comparisons using the Bonferroni method revealed that individuals with moderate and severe communication anxiety had higher rates of mobile phone dependence. In addition, individuals who spent 5–9.9 h per day using their mobile phone also had higher rates of mobile phone dependence. Detailed results are shown in Table 2.

**Table 2:** Univariate analysis of demographic characteristics in relation to mobile phone dependence

Demographic characteristics	Mobile phone dependence status		Mobile phone dependence rate (%)	χ^2^	*P*
	Yes	No			
Gender				1.709	0.191
Male	163	77	67.9		
Female	192	115	62.5		
Major				0.473	0.492
Clinical medicine	319	176	64.4		
Non-clinical medicine	36	16	69.2		
Residential location of household				1.841	0.175
Urban area	237	139	63.0		
Rural area	118	53	69.0		
Monthly living expenses (Yuan)				0.922	0.631
< 1000	29	20	59.2		
1000–1999	257	133	65.9		
≥ 2000	69	39	63.9		
Parental marital status				0.346	0.556
Primary marriage	316	174	64.5		
Remarried or divorced	39	18	68.4		
Only child status				0.013	0.909
Yes	78	43	64.5		
No	277	149	65.0		
Current romantic relationship status				0.622	0.430
Yes	37	16	69.8		
No	318	176	64.4		
Communication anxiety				35.135	< 0.001
Mild	66	80	45.2		
Moderate	257	104	71.2^a^		
Severe	32	8	80.0^a^		
The duration of mobile phone use (h)				29.620	< 0.001
1-4.9	95	96	49.7		
5-9.9	215	79	73.1^#^		
≥ 10	45	17	72.6^#^		

^a^Compared to those with mild anxiety, individuals with moderate and severe communication anxiety had higher rates of mobile phone dependence

#Compared to those who use mobile phones for 1–4.9 h per day, those who use mobile phones for ≥5 h per day had higher rates of mobile phone dependence

### Rank correlation of mental health literacy and mobile phone dependence among college students

Spearman’s rank correlation analysis was performed between mental health literacy and mobile phone dependence among college students. The results indicated that there was no significant correlation between mobile phone dependence and the total score of mental health literacy (*P* > 0.05). Mobile phone dependence and communication anxiety scores were significantly correlated (*P* < 0.05). Detailed results are shown in Table 3.

**Table 3:** Correlation analysis between mental health literacy and mobile phone dependence among college students

Variable	1 Mental health literacy	2 Mobile phone dependence	3 Communication anxiety
1 Mental health literacy	1		
2 Mobile phone dependence	-0.036	1	
3 Communication anxiety	0.015	0.354^**^	1

**P < 0.05

### Analysis of factors associated with mobile phone dependence among college students

A hierarchical regression analysis was conducted to examine the factors associated with mobile phone dependence among first-year college students. The total score of mobile phone dependence was taken as the dependent variable. Personal data regarding the students—namely, gender, major, family residence, monthly living expenses, the duration of mobile phone use, parental marital status, only-child status, and romantic relationship status—were included as control variables. Total scores of mental health literacy and communication anxiety were entered into the regression equation as independent variables in sequential steps. To evaluate the proposed moderating role of mental health literacy, an interaction term (mental health literacy × communication anxiety) was entered in the final step of the hierarchical regression model.

Model 3 accounted for 16.70% of the variance in the dependent variable (*R*^*2*^ = 0.167, *F* = 8.904, *P* < 0.001). The addition of communication anxiety in Model 3 significantly increased the explained variance of mobile phone dependence (△*R*^*2*^ = 0.141, *F* = 90.505,*P* < 0.001). These results indicate a stronger association between communication anxiety and mobile phone dependence than between mental health literacy and mobile phone dependence. After controlling for demographic characteristics and mental health literacy among first-year students, monthly living expenses and communication anxiety were associated with mobile phone dependence (*P* < 0.001). The standardized regression coefficient for communication anxiety was *β* = 0.380, making it the strongest correlate of mobile phone dependence among the variables included in the model.

To test Hypothesis 3, the interaction term between mental health literacy and communication anxiety was entered in Model 4. The interaction term was not statistically significant (*B* = 0.002, *β* = 0.148, *t* = 0.350, *P* = 0.727), and its inclusion did not increase the explained variance of the model (△*R*²=0.000, *P* = 0.727). Therefore, Hypothesis 3 was not supported, indicating that mental health literacy did not significantly moderate the association between communication anxiety and mobile phone dependence. Detailed results are shown in Table 4.

**Table 4:** Hierarchical regression results for mobile phone dependence among college students

Variable	Model 1				Model 2				Model 3				Model 4			
	B	β	t	*P*	B	β	t	*P*	B	β	t	*P*	B	β	t	*P*
Gender	0.623	0.032	0.724	0.469	0.628	0.032	0.729	0.466	0.772	0.039	0.969	0.333	0.776	0.040	0.972	0.331
Major	-0.748	-0.023	-0.525	0.600	-0.760	-0.023	-0.532	0.595	-0.846	-0.025	-0.640	0.522	-0.824	-0.025	-0.622	0.534
Residential location of household	-0.029	-0.001	-0.030	0.976	-0.039	-0.002	-0.04	0.968	0.308	0.015	0.343	0.732	0.306	0.015	0.340	0.734
Monthly living expenses (Yuan)																
1000–1999	3.613	0.168	2.364	0.018	3.609	0.168	2.359	0.019	3.409	0.158	2.407	0.016	3.423	0.159	2.415	0.016
≥ 2000	4.404	0.180	2.519	0.012	4.416	0.181	2.521	0.012	4.414	0.181	2.723	0.007	4.431	0.181	2.730	0.007
The duration of mobile phone use (h)																
5-9.9	1.660	0.085	1.805	0.072	1.660	0.085	1.803	0.072	1.536	0.079	1.802	0.072	1.533	0.078	1.796	0.073
≥ 10	1.491	0.049	1.040	0.299	1.494	0.049	1.041	0.299	1.198	0.039	0.902	0.368	1.236	0.040	0.927	0.355
Parental marital status	0.620	0.019	0.440	0.660	0.628	0.020	0.446	0.656	-0.174	-0.005	-0.133	0.894	-0.168	-0.005	-0.128	0.898
Only child status	0.557	0.024	0.521	0.603	0.563	0.024	0.526	0.599	0.237	0.010	0.239	0.811	0.247	0.011	0.249	0.803
Current romantic relationship status	0.214	0.007	0.152	0.879	0.215	0.007	0.152	0.879	-1.281	-0.039	-0.972	0.332	-1.263	-0.038	-0.956	0.339
Mental health literacy		-	-	-	-0.009	-0.007	-0.155	0.877	-0.036	-0.027	-0.687	0.492	-0.129	-0.099	-0.475	0.635
Communication anxiousness		-	-	-		-	-	-	0.458	0.380	9.513	< 0.001	0.308	0.255	0.710	0.478
Mental health literacy×Communication anxiousness	-	-	-	-	-	-	-	-	-	-	-	-	0.002	0.148	0.350	0.727
* R* ^*2*^	0.025				0.025				0.167				0.167			
* F*	1.400				1.272				8.904				8.215			
* P*	0.177				0.237				< 0.001				< 0.001			
△*R*^*2*^	0.025				0.000				0.141				0.000			
* F*	1.400				0.024				90.505				0.122			
* P*	0.177				0.877				< 0.001				0.727			

Reference categories were as follows: male (gender), clinical medicine (major), rural area (family residence), < 1000 Yuan (monthly living expenses), 1–4.9 h (duration of mobile phone use), first marriage (parental marital status), yes (only-child status), and yes (current romantic relationship status)

## Discussion

The vigorous rise of the internet and the popularization of mobile phones have led to the deep integration of these technologies into people’s daily lives. Communication technology is ubiquitous on university campuses, where mobile phone dependence is particularly prominent. For students just entering college, excessive use of mobile phones may hinder their academic progress, and it may have adverse effects on physical and mental health. In view of this, it is particularly urgent to conduct research on the problem of mobile phone dependence among first-year university students [33, 34].

The rate of mobile phone dependence among college students in our study was 64.9%—higher than the result obtained by Zhang et al. [35] (49.5%) and similar to that of Alotaibi et al. [36] (67%). Our study found that monthly living expenses and communication anxiety were associated with mobile phone dependence. Specifically, in terms of monthly living expenses, the study found that higher monthly living expenses were associated with a greater likelihood of mobile phone dependence among college students, which was consistent with the research results of Giraldo-Jimenez et al. [37]. There are at least two possible reasons for this. First, college students with high monthly living expenses are better able to purchase high-performance mobile phones. In addition to basic calling and messaging functions, such mobile phones also cater to online shopping, ticket purchasing, take-out food ordering, and other functions, making mobile phones an indispensable part of the lives of first-year college students. This may be associated with a higher frequency of mobile phone use and a greater likelihood of mobile phone dependence [38, 39]. Second, first-year students from families with different economic levels have different ages of mobile phone ownership. Students from more affluent families report earlier ages of mobile phone use, longer years of mobile phone use, and are more likely to have mobile phone dependence [40, 41]. Higher monthly living expenses may also provide students with greater opportunities for digital consumption [42]. Students with more financial resources may participate more frequently in online entertainment, social networking, and other digital activities, which could increase both the frequency and duration of mobile phone use [43]. Thus, the relationship between monthly living expenses and mobile phone dependence may reflect broader behavioral and lifestyle factors rather than purely technological factors.

In addition, we found that higher rates of mobile phone dependence among college students were associated with high levels of communication anxiety, consistent with the findings of Zhou et al. [44]. According to the theory of compensatory internet use, when people encounter psychological problems in reality, they may resort to the internet or mobile phones as a form of escapism [45]. Students with severe communication anxiety may experience difficulties in interpersonal relationships and perceive lower levels of social support in real life, and the colorful functions and virtual world of mobile phones provide them with an outlet to divert their attention and temporarily forget their real-world social problems [46]. Mobile phones may provide temporary comfort for individuals with communication anxiety, which may be associated with more frequent smartphone use and greater reliance on online interaction. Tao et al. [47] found that mobile phone dependence can exacerbate psychological problems such as communication anxiety, thus forming a vicious circle of mobile phone dependence.

Two points do not fit with our assumptions. The first is that no correlation was found between mobile phone dependence and the total score of mental health literacy, indicating that mobile phone dependence cannot be simply attributed to an individual’s level of mental health literacy, and that the association between mental health literacy and mobile phone dependence may be affected by the key link of cognitive transformation into behavior. Mental health literacy belongs to the literacy of individual cognition, which is mainly reflected in the mastery of mental health knowledge and cognitive attitude towards mental health problems. As an addictive behavior, the formation of mobile phone dependence depends more on the self-regulation and emotional control ability at the behavior level [48]. Previous studies have shown that emotional regulation deficits often mediate the relationship between psychological problems and smartphone use problems, while the key factor of addictive behavior is the self-regulation process [49, 50]. This means that even if individuals have a full understanding of mental health knowledge and an open attitude toward mental health problems, it does not necessarily translate into actual behavior change or self-regulation ability. Future research should further consider emotion regulation, self-control, coping strategies, and social support alongside mental health literacy to better understand the psychological and social correlates of smartphone use behaviors. Furthermore, the causes of mobile phone dependence are complex and diverse. They relate not only to mental health literacy but also to age, personality, living environment, social support networks, and coping strategies [51, 52]. For example, some individuals may use mobile phones excessively as a way to manage psychological needs such as loneliness, escapism, and a desire to seek immediate gratification, which may not depend directly on their level of knowledge about mental health or their views on psychological issues. The absence of the expected association between mobile phone dependence and mental health literacy in this study does not indicate that mental health literacy has no effect on mobile phone dependence, but suggests that the association may not be directly linear. Mental health literacy may be indirectly associated with mobile phone dependence through intermediate variables such as self-regulation. This finding also provides a useful direction for future research to further explore potential mediating mechanisms underlying the association between mental health literacy and mobile phone dependence.

The second is that the interaction between mental health literacy and communication anxiety was not detected. One possible explanation is that mental health literacy primarily reflects cognitive resources, whereas communication anxiety may be more closely related to emotional and behavioral responses in social situations [53–55]. As a relatively stable psychological resource, the potential buffering effect of mental health literacy may not be substantially influenced by the level of individual communication anxiety [56, 57]. Individuals with different levels of communication anxiety may potentially benefit from higher levels of mental health literacy. Communication anxiety may be associated with greater psychological distress and a tendency to compensate through online social and entertainment activities [58, 59]. This pathway mainly relies on emotional regulation motivation. Together, these differences may help explain why no significant interaction effect was observed in the present study. These findings may have implications for future intervention research.

### Limitations

This study has limitations. First, the survey scope was restricted to one university, and the representation of college students was limited. Second, our study population only included first-year students, and changes in mobile phone dependence and mental health literacy could not be tracked. Third, the effect of mental health literacy may be delayed. Naturally, we were only able to examine the relationship between mobile phone dependence and mental health literacy from the perspective of a cross-sectional study. Therefore, a follow-up study could be carried out involving a larger sample, and the population of college students could be extended from medical colleges to other comprehensive colleges. In addition, a follow-up study could be designed to evaluate whether the incidence of mobile phone dependence increases among college students with different levels of mental health literacy after their senior year. Fourth, the categorical treatment of continuous variables, such as daily hours of mobile phone use and monthly living expenses, may have reduced statistical power and introduced subjective cutoff values. Furthermore, the relatively low *R*² value (16.7%) indicates that a substantial proportion of the variability in mobile phone dependence remained unexplained. This finding suggests that mobile phone dependence may be associated with a broad range of individual, social, and environmental factors beyond those examined in the present study. Fifth, this study found that the scores of the mental health literacy scale were not normally distributed, which may be an accidental feature of the study sample, and the distribution of mental health literacy in this sample may not be representative of the population. Future studies can be validated using probability sampling or larger samples. Sixth, this study mainly focused on young people, and future studies are needed to verify whether the results are applicable to other age groups. All variables were assessed via self-report, which may be subject to recall bias, response bias, and social desirability bias—particularly for mobile phone dependence, communication anxiety, and mental health literacy. Future research should consider objective behavioral measures or multi-method assessments to reduce these biases.

## Conclusion

This study found that monthly living expenses and communication anxiety were associated with mobile phone dependence among first-year college students, whereas mental health literacy was not significantly associated with mobile phone dependence. In addition, mental health literacy did not significantly moderate the association between communication anxiety and mobile phone dependence. These findings suggest that communication anxiety may be more closely associated with mobile phone dependence than mental health literacy among first-year college students. Therefore, future research and intervention programs addressing mobile phone dependence may benefit from paying greater attention to communication anxiety and related psychosocial factors. Further longitudinal and multi-center studies are needed to clarify the underlying mechanisms and explore potential indirect pathways linking mental health literacy and mobile phone dependence.

## Supplementary Information

Supplementary Material 1: MHLq_SVa. Mental Health Literacy questionnaire – short version adult. Smartphone Addiction Scale – Short Version,SAS-SV. Interaction Anxiousness Scale, IAS.

## Data Availability

The dataset record is deposited in ScienceDB at 10.57760/sciencedb.28893. The raw data are not publicly available due to privacy and ethical restrictions, but may be made available from the corresponding author upon reasonable request. Requests for data access should be directed to Si-yang YE, Guangdong Medical University, at agito@gdmu.edu.cn.
