# Supplementary material for: Correlation between mental health literacy, communication anxiety, and mobile phone dependence among first-year college students
Source: BMC Public Health. 2026 Jul 20;26:3279. doi: 10.1186/s12889-026-28594-5 (PMC13749774; doi:10.1186/s12889-026-28594-5)
Supplement: Supplementary file 1 [file 12889_2026_28594_MOESM1_ESM.docx]

**MHLq_SVa**

**Mental Health Literacy questionnaire – short version adult**

Campos, Dias, Costa, Rabin, Miles, Lestari, Feraihan, Pant, Sriwichai, Boonchieng, & Yu (2022)

The following questions focus on aspects related to mental health issues, such as what you think about this type of problems, which resources people may look for to get help. For each statement, please tick the option that indicates how much you agree or disagree. Please answer honestly; do not worry about giving the “right” answer.

|  | **Strongly disagree** | **Disagree** | **Neither agree nor disagree** | **Agree** | **Strongly agree** |
| --- | --- | --- | --- | --- | --- |
| 1.Physical exercise contributes to a good mental health. |  |  |  |  |  |
| 2. Mental disorders don’t affect people’s behaviours. |  |  |  |  |  |
| 3. Sleeping well contributes to a good mental health. |  |  |  |  |  |
| 4. If I had a mental disorder I would seek for a psychologist’s help. |  |  |  |  |  |
| 5. Mental disorders don’t affect people’s feelings. |  |  |  |  |  |
| 6. Only adults have mental disorders. |  |  |  |  |  |
| 7. Changes in brain function may lead to the onset of mental. |  |  |  |  |  |
| 8. If someone close to me had a mental disorder, I would encourage her/him to see a psychiatrist. |  |  |  |  |  |
| 9. A balanced diet contributes to good mental health. |  |  |  |  |  |
| 10. One of the symptoms of depression is the loss of interest or pleasure in most things. |  |  |  |  |  |
| 11. The symptom’s length is one of the important criteria for the diagnosis of a mental disorder. |  |  |  |  |  |
| 12. Mental disorders affect people’s thoughts. |  |  |  |  |  |
| 13. Doing something enjoyable contributes to a good mental health. |  |  |  |  |  |
| 14. A person with schizophrenia may see and hear things that nobody else sees and hears. |  |  |  |  |  |
| 15. Highly stressful situations may cause mental disorders. |  |  |  |  |  |
| 16. If I had a mental disorder, I would seek for a psychiatrist’s help. |  |  |  |  |  |

**Smartphone Addiction Scale – Short Version,SAS-SV**

The following statements describe mobile phone usage. Please check "√" the option that best matches your actual situation.

|  | **Strongly disagree** | **Disagree** | **Slightly disagree** | **Slightly agree** | **Agree** | **Strongly agree** |
| --- | --- | --- | --- | --- | --- | --- |
| 1.Missing planned work due to smartphone use. |  |  |  |  |  |  |
| 2.Having a hard time concentrating in class, while doing assignments, or while working due tosmartphone use. |  |  |  |  |  |  |
| 3.Feeling pain in the wrists or at the back of the neck while using a smartphone. |  |  |  |  |  |  |
| 4.Won't be able to stand not having a smartphone. |  |  |  |  |  |  |
| 5.Feeling impatient and fretful when l am not holding my smartphone. |  |  |  |  |  |  |
| 6.Having my smartphone in my mind even when l am not using it. |  |  |  |  |  |  |
| 7.Iwill never give up using my smartphone even when my daily life is already greatly affected by it. |  |  |  |  |  |  |
| 8.Constantly checking my smartphone so as not to miss conversations between other people on Twitter or Facebook. |  |  |  |  |  |  |
| 9.Using my smartphone longer than l had intended. |  |  |  |  |  |  |
| 10.The people around me tell me that l use my smartphone too much. |  |  |  |  |  |  |

**Interaction Anxiousness Scale, IAS**

Please recall your interactions with others over the past two weeks. Select the option that best describes your feelings and check "√" in the corresponding box.

|  | Does not apply to me at all | Applies to me a little | Applies to me moderately | Applies to me very much | Applies to me completely |
| --- | --- | --- | --- | --- | --- |
| 1.I often feel nervous even in casual get-togethers. |  |  |  |  |  |
| 2.I usually feel uncomfortable when I am in a group of people I don't know. |  |  |  |  |  |
| 3.I am usually at ease when speaking to a member of the opposite sex.* |  |  |  |  |  |
| 4.I get nervous when I must talk to a teacher or boss. |  |  |  |  |  |
| 5.Parties often make me feel anxious and uncomfortable. |  |  |  |  |  |
| 6.I am probably less shy in social interactions than most people.* |  |  |  |  |  |
| 7.I sometimes feel tense when talking to people of my own sex if I don't know them very well. |  |  |  |  |  |
| 8.I would be nervous if I was being interviewed for a job. |  |  |  |  |  |
| 9.I wish I had more confidence in social situations. |  |  |  |  |  |
| 10.I seldom feel anxious in social situations.* |  |  |  |  |  |
| 11.In general, I am a shy person. |  |  |  |  |  |
| 12.I often feel nervous when talking to an attractive member of the opposite sex. |  |  |  |  |  |
| 13.I often feel nervaus when calling someone I don't know very well on the telephone. |  |  |  |  |  |
| 14.I get nervous when I speak to someone in a position of authority. |  |  |  |  |  |
| 15.I usually feel relaxed around other people, even people who are quite different from me.* |  |  |  |  |  |

Note:Items marked with an asterisk (*) are reverse-scored.
